# (*E*)-1-[2-(Methyl­sulfan­yl)phen­yl]-2-({(*E*)-2-[2-(methyl­sulfan­yl)phen­yl]hydrazinyl­idene}(nitro)­meth­yl)diazene

**DOI:** 10.1107/S1600536811054080

**Published:** 2011-12-21

**Authors:** Karel G. von Eschwege, Fabian Muller, Eric C. Hosten

**Affiliations:** a Department of Chemistry, University of the Free State, PO Box 339, Bloemfontein 9300, South Africa; b Department of Chemistry, Nelson Mandela Metropolitan University, PO Box 77000, Port Elizabeth 6031, South Africa

## Abstract

In the title compound, C_15_H_15_N_5_O_2_S_2_, the phenyl rings make dihedral angles of 4.03 (4) and 9.77 (5)° with the plane defined by the central N—N—C—N—N atoms (r.m.s. deviation = 0.010 Å). The C—S—C—C torsion angles of the methyl­sulfanyl groups with their respective phenyl rings are −7.47 (13) and −72.07 (13)°. The shortest centroid–centroid distance of 3.707 Å occurs between the two π-systems N—N—C—N—N and the benzene ring in the diazene 1-position. The H atom bound to the N atom is involved in intra­molecular N—H⋯N and N—H⋯S contacts, while the nitro O atoms are involved in inter­molecular C—H⋯O contacts.

## Related literature

For the chemistry of dithizone, see: Irving (1977 ▶). For related structures, see: Laing (1977 ▶); Mito *et al.* (1997 ▶); Gilroy *et al.* (2008 ▶). For the synthesis of nitro­formazans, see: Pelkis *et al.* (1957 ▶). For DFT and electrochemistry studies of dithizone, see: von Eschwege & Swarts (2010 ▶); von Eschwege, Conradie & Kuhn (2011 ▶).
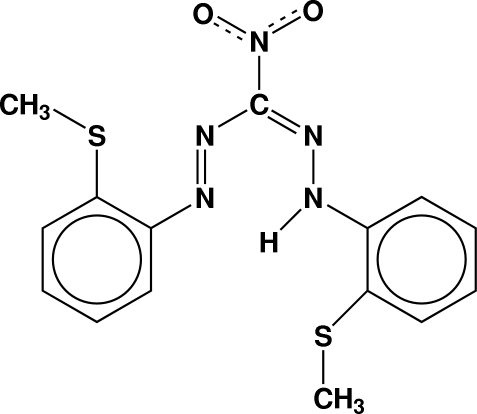


## Experimental

### 

#### Crystal data

C_15_H_15_N_5_O_2_S_2_
*M* *_r_* = 361.44
Monoclinic, 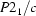

*a* = 4.7283 (2) Å
*b* = 17.9791 (10) Å
*c* = 19.3865 (8) Å
β = 103.646 (2)°
*V* = 1601.54 (13) Å^3^
*Z* = 4
Mo *K*α radiation
μ = 0.35 mm^−1^
*T* = 200 K
0.79 × 0.21 × 0.07 mm

#### Data collection

Bruker APEXII CCD diffractometer
Absorption correction: multi-scan (*SADABS*; Bruker, 2008 ▶) *T*_min_ = 0.870, *T*_max_ = 1.000
14965 measured reflections
3960 independent reflections
3301 reflections with *I* > 2σ(*I*)
*R*_int_ = 0.019

#### Refinement

*R*[*F* ^2^ > 2σ(*F* ^2^)] = 0.031
*wR*(*F* ^2^) = 0.086
*S* = 1.04
3960 reflections
219 parameters
H-atom parameters constrained
Δρ_max_ = 0.31 e Å^−3^
Δρ_min_ = −0.25 e Å^−3^

### 

Data collection: *APEX2* (Bruker, 2010 ▶); cell refinement: *SAINT* (Bruker, 2010 ▶); data reduction: *SAINT*; program(s) used to solve structure: *SHELXS97* (Sheldrick, 2008 ▶); program(s) used to refine structure: *SHELXL97* (Sheldrick, 2008 ▶); molecular graphics: *ORTEP-3* (Farrugia, 1997 ▶) and *Mercury* (Macrae *et al.*, 2006 ▶); software used to prepare material for publication: *SHELXL97*, *PLATON* (Spek, 2009 ▶) and *publCIF* (Westrip, 2010 ▶).

## Supplementary Material

Crystal structure: contains datablock(s) I, global. DOI: 10.1107/S1600536811054080/zq2147sup1.cif

Structure factors: contains datablock(s) I. DOI: 10.1107/S1600536811054080/zq2147Isup2.hkl

Supplementary material file. DOI: 10.1107/S1600536811054080/zq2147Isup3.cml

Additional supplementary materials:  crystallographic information; 3D view; checkCIF report

## Figures and Tables

**Table 1:** Hydrogen-bond geometry (Å, °)

*D*—H⋯*A*	*D*—H	H⋯*A*	*D*⋯*A*	*D*—H⋯*A*
N4—H4⋯S2	0.88	2.60	3.0248 (13)	110
N4—H4⋯N1	0.88	1.99	2.6229 (16)	128
C2—H2*B*⋯O1^i^	0.98	2.36	3.253 (2)	151
C25—H25⋯O2^ii^	0.95	2.45	3.1901 (19)	134

Symmetry codes: (i) 
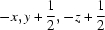
; (ii) 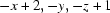.

**Fig. 1.**
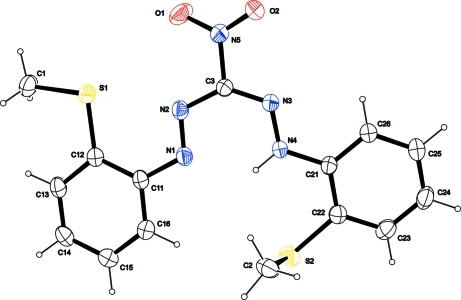
The molecular structure of the title compound, with atom labels and anistropic displacement ellipsoids drawn at the 50% probability level.

**Fig. 2.**
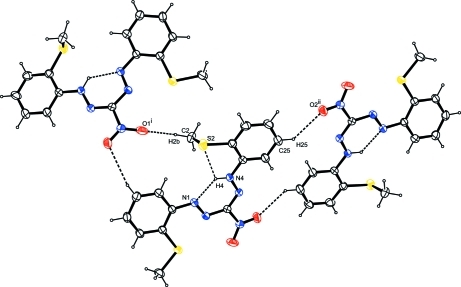
Inter- and intramolecular contacts in the crystal structure of the title compound (ellipsoids drawn at the 50% probability level). Symmetry operators: (i) = -x, y + 1/2, -z + 1/2; (ii) = -x + 2, -y, -z + 1.

**Fig. 3.**
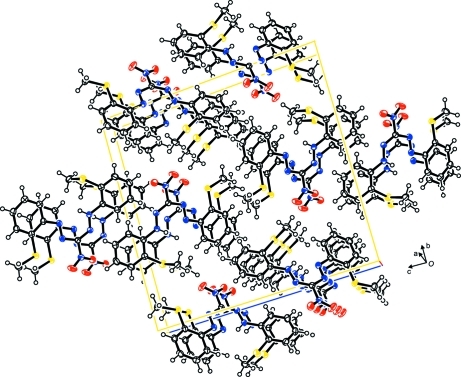
Molecular packing of the title compound (anistropic displacement ellipsoids drawn at 50% probability level).

## supplementary crystallographic information

### Comment

During the synthesis of the versatile trace metal analysis dithizone reagent, aniline is first diazotized and then treated with nitromethane to form the bright orange-red nitroformazan product (Pelkis *et al.*, 1957). Ammonia and hydrogen sulfide gas are used to substitute the nitro group with sulfur towards the formation of dithizone, the chemistry of which is extensively described in the literature (Irving, 1977). Single crystal X-ray structures of nitroformazan derivatives were determined by Gilroy *et al.* (2008), Mito *et al.* (1997) and the dithizone structure by Laing (1977), while we performed extensive DFT (von Eschwege *et al.*, 2011) and electrochemistry studies (von Eschwege & Swarts, 2010) on the free ligand.

We recently embarked on a study during which we synthesized a series of electronically altered dithizones for the purpose of investigating its altered redox and structural properties. During this process, crystals of the title compound, suitable for X-ray crystallography, were grown from an acetone solution overlaid with *n*-hexane.

The least square planes defined by the phenyl rings with respect to the plane defined by the N1, N2, C3, N3 and N4 atoms enclose dihedral angles of 9.77 (5)° and 4.03 (4)° (Fig. 1). The torsion angles of the *S*-methyl groups with their respective phenyl rings are 7.47 (13)° and 72.07 (13)°. The shortest centroid-centroid distance of 3.707 Å occurs between the two π-systems N1—N2—C3—N3—N4 and C11—C12—C13—C14—C15—C16. The H atom bound to N4 is involved in intramolecular N—H···N and N—H···S contacts while the nitro O atoms have intermolecular C—H···O contacts (Fig. 2). The packing of the title compound in the crystal is shown in Figure 3.

### Experimental

Solvents (AR) purchased from Merck and reagents from Sigma-Aldrich were used without further purification. The *ortho*-*S*-methyl derivative of nitroformazan was prepared according to the procedure reported by Pelkis *et al*. (1957). *M*.p. 144 °C. λ_max_ (dichloromethane) 320, 479 nm. δ_H_ (600 MHz, CDCl_3_) 14.76 (1 H, 1 × s, 1 × NH), 2.50 (6 H, 1 × s, 2 × SCH_3_), 8.03 – 7.34 (8 H, 3 × m, 2 × C_6_H_4_)

### Refinement

All hydrogen positions were calculated after each cycle of refinement using a riding model, with C—H = 0.95 Å and *U*_iso_(H) = 1.2*U*_eq_(C) for aromatic H atoms, with N—H = 0.88 Å and *U*_iso_(H) = 1.2*U*_eq_(N), and with C—H = 0.98 Å and *U*_iso_(H) = 1.5*U*_eq_(C) for methyl H atoms. The H atoms of the methyl groups were allowed to rotate with a fixed angle around the C—C bond to best fit the experimental electron density [HFIX 137 in *SHELXL97* (Sheldrick, 2008)].

### Crystal data

**Table:**

C_15_H_15_N_5_O_2_S_2_	*F*(000) = 752
*M**_r_* = 361.44	*D*_x_ = 1.499 Mg m^−^^3^
Monoclinic, *P*2_1_/*c*	Melting point: 417 K
Hall symbol: -P 2ybc	Mo *K*α radiation, λ = 0.71073 Å
*a* = 4.7283 (2) Å	Cell parameters from 8473 reflections
*b* = 17.9791 (10) Å	θ = 2.3–28.3°
*c* = 19.3865 (8) Å	µ = 0.35 mm^−^^1^
β = 103.646 (2)°	*T* = 200 K
*V* = 1601.54 (13) Å^3^	Platelet, red
*Z* = 4	0.79 × 0.21 × 0.07 mm

### Data collection

**Table:**

Bruker APEXII CCD diffractometer	3960 independent reflections
Radiation source: sealed tube	3301 reflections with *I* > 2σ(*I*)
graphite	*R*_int_ = 0.019
φ and ω scans	θ_max_ = 28.3°, θ_min_ = 2.2°
Absorption correction: multi-scan (*SADABS*; Bruker, 2008)	*h* = −6→5
*T*_min_ = 0.870, *T*_max_ = 1.000	*k* = −23→23
14965 measured reflections	*l* = −25→25

### Refinement

**Table:**

Refinement on *F*^2^	Primary atom site location: structure-invariant direct methods
Least-squares matrix: full	Secondary atom site location: difference Fourier map
*R*[*F*^2^ > 2σ(*F*^2^)] = 0.031	Hydrogen site location: inferred from neighbouring sites
*wR*(*F*^2^) = 0.086	H-atom parameters constrained
*S* = 1.04	*w* = 1/[σ^2^(*F*_o_^2^) + (0.0396*P*)^2^ + 0.7279*P*] where *P* = (*F*_o_^2^ + 2*F*_c_^2^)/3
3960 reflections	(Δ/σ)_max_ < 0.001
219 parameters	Δρ_max_ = 0.31 e Å^−^^3^
0 restraints	Δρ_min_ = −0.25 e Å^−^^3^

### Special details

**Table:**

Geometry. All e.s.d.'s (except the e.s.d. in the dihedral angle between two l.s. planes) are estimated using the full covariance matrix. The cell e.s.d.'s are taken into account individually in the estimation of e.s.d.'s in distances, angles and torsion angles; correlations between e.s.d.'s in cell parameters are only used when they are defined by crystal symmetry. An approximate (isotropic) treatment of cell e.s.d.'s is used for estimating e.s.d.'s involving l.s. planes.
Refinement. Refinement of *F*^2^ against ALL reflections. The weighted *R*-factor *wR* and goodness of fit *S* are based on *F*^2^, conventional *R*-factors *R* are based on *F*, with *F* set to zero for negative *F*^2^. The threshold expression of *F*^2^ > σ(*F*^2^) is used only for calculating *R*-factors(gt) *etc*. and is not relevant to the choice of reflections for refinement. *R*-factors based on *F*^2^ are statistically about twice as large as those based on *F*, and *R*- factors based on ALL data will be even larger.

### Fractional atomic coordinates and isotropic or equivalent isotropic displacement parameters (Å^2^)

**Table:**

	*x*	*y*	*z*	*U*_iso_*/*U*_eq_	
S1	−0.33810 (7)	−0.05295 (2)	0.125542 (19)	0.02859 (10)	
S2	0.10204 (8)	0.22354 (2)	0.39420 (2)	0.03395 (11)	
O1	0.2660 (3)	−0.13599 (7)	0.23524 (7)	0.0451 (3)	
O2	0.6661 (3)	−0.11093 (7)	0.31138 (6)	0.0419 (3)	
N1	−0.0436 (2)	0.06137 (7)	0.24775 (6)	0.0261 (2)	
N2	0.0632 (2)	−0.00197 (7)	0.23768 (6)	0.0254 (2)	
N3	0.4414 (3)	0.00974 (7)	0.34898 (6)	0.0262 (2)	
N4	0.3493 (3)	0.07389 (7)	0.36783 (6)	0.0275 (3)	
H4	0.2011	0.0966	0.3397	0.033*	
N5	0.4207 (3)	−0.09471 (7)	0.27813 (6)	0.0288 (3)	
C1	−0.6361 (3)	−0.08689 (10)	0.05684 (8)	0.0368 (3)	
H1A	−0.8170	−0.0839	0.0731	0.055*	
H1B	−0.5993	−0.1387	0.0460	0.055*	
H1C	−0.6541	−0.0565	0.0141	0.055*	
C2	0.2794 (4)	0.25326 (9)	0.32603 (9)	0.0364 (3)	
H2A	0.4660	0.2766	0.3481	0.055*	
H2B	0.1553	0.2892	0.2948	0.055*	
H2C	0.3132	0.2101	0.2982	0.055*	
C3	0.3021 (3)	−0.02142 (8)	0.29019 (7)	0.0244 (3)	
C11	−0.2889 (3)	0.08284 (8)	0.19544 (7)	0.0242 (3)	
C12	−0.4468 (3)	0.03812 (8)	0.13894 (7)	0.0241 (3)	
C13	−0.6941 (3)	0.07045 (9)	0.09478 (8)	0.0292 (3)	
H13	−0.8071	0.0420	0.0569	0.035*	
C14	−0.7781 (3)	0.14243 (9)	0.10470 (8)	0.0326 (3)	
H14	−0.9475	0.1623	0.0737	0.039*	
C15	−0.6196 (3)	0.18619 (9)	0.15909 (8)	0.0321 (3)	
H15	−0.6756	0.2361	0.1650	0.039*	
C16	−0.3795 (3)	0.15564 (8)	0.20428 (8)	0.0292 (3)	
H16	−0.2721	0.1847	0.2425	0.035*	
C21	0.4883 (3)	0.10600 (8)	0.43295 (7)	0.0259 (3)	
C22	0.3908 (3)	0.17503 (8)	0.45127 (7)	0.0264 (3)	
C23	0.5239 (3)	0.20576 (9)	0.51675 (8)	0.0328 (3)	
H23	0.4580	0.2523	0.5301	0.039*	
C24	0.7501 (4)	0.16978 (10)	0.56265 (8)	0.0364 (3)	
H24	0.8379	0.1913	0.6073	0.044*	
C25	0.8481 (4)	0.10248 (10)	0.54336 (9)	0.0400 (4)	
H25	1.0057	0.0780	0.5746	0.048*	
C26	0.7182 (4)	0.07032 (9)	0.47872 (8)	0.0371 (4)	
H26	0.7863	0.0239	0.4657	0.045*	

### Atomic displacement parameters (Å^2^)

**Table:**

	*U*^11^	*U*^22^	*U*^33^	*U*^12^	*U*^13^	*U*^23^
S1	0.02626 (18)	0.02805 (19)	0.02859 (18)	−0.00116 (13)	0.00074 (13)	−0.00361 (13)
S2	0.02892 (19)	0.0374 (2)	0.0351 (2)	0.01037 (15)	0.00660 (14)	−0.00065 (15)
O1	0.0362 (6)	0.0348 (6)	0.0586 (8)	−0.0037 (5)	−0.0001 (5)	−0.0214 (6)
O2	0.0419 (6)	0.0366 (6)	0.0381 (6)	0.0139 (5)	−0.0090 (5)	−0.0063 (5)
N1	0.0245 (6)	0.0282 (6)	0.0231 (5)	−0.0008 (4)	0.0008 (4)	0.0003 (5)
N2	0.0238 (5)	0.0268 (6)	0.0234 (5)	−0.0031 (4)	0.0017 (4)	0.0001 (4)
N3	0.0288 (6)	0.0227 (6)	0.0240 (5)	0.0000 (4)	0.0003 (4)	−0.0006 (4)
N4	0.0288 (6)	0.0243 (6)	0.0244 (6)	0.0029 (5)	−0.0037 (4)	−0.0018 (5)
N5	0.0322 (6)	0.0251 (6)	0.0273 (6)	−0.0010 (5)	0.0034 (5)	−0.0023 (5)
C1	0.0343 (8)	0.0377 (9)	0.0340 (8)	−0.0035 (6)	−0.0008 (6)	−0.0093 (7)
C2	0.0401 (8)	0.0297 (8)	0.0382 (8)	0.0071 (6)	0.0068 (7)	0.0080 (6)
C3	0.0261 (6)	0.0221 (7)	0.0235 (6)	−0.0010 (5)	0.0024 (5)	−0.0001 (5)
C11	0.0214 (6)	0.0281 (7)	0.0217 (6)	−0.0020 (5)	0.0026 (5)	0.0018 (5)
C12	0.0225 (6)	0.0274 (7)	0.0223 (6)	−0.0025 (5)	0.0052 (5)	0.0011 (5)
C13	0.0251 (7)	0.0344 (8)	0.0249 (6)	−0.0026 (5)	−0.0003 (5)	0.0013 (6)
C14	0.0286 (7)	0.0358 (8)	0.0302 (7)	0.0039 (6)	0.0006 (5)	0.0066 (6)
C15	0.0333 (8)	0.0290 (8)	0.0330 (7)	0.0043 (6)	0.0056 (6)	0.0028 (6)
C16	0.0294 (7)	0.0298 (8)	0.0266 (6)	−0.0012 (6)	0.0032 (5)	−0.0025 (6)
C21	0.0263 (7)	0.0252 (7)	0.0235 (6)	−0.0021 (5)	0.0003 (5)	−0.0014 (5)
C22	0.0265 (7)	0.0278 (7)	0.0248 (6)	−0.0009 (5)	0.0057 (5)	−0.0001 (5)
C23	0.0393 (8)	0.0304 (8)	0.0295 (7)	−0.0020 (6)	0.0098 (6)	−0.0060 (6)
C24	0.0430 (9)	0.0376 (9)	0.0248 (7)	−0.0078 (7)	0.0002 (6)	−0.0059 (6)
C25	0.0422 (9)	0.0386 (9)	0.0303 (7)	0.0031 (7)	−0.0090 (6)	−0.0022 (7)
C26	0.0413 (8)	0.0301 (8)	0.0316 (8)	0.0070 (6)	−0.0080 (6)	−0.0051 (6)

### Geometric parameters (Å, °)

**Table:**

S1—C12	1.7538 (15)	C11—C16	1.400 (2)
S1—C1	1.8018 (15)	C11—C12	1.4205 (18)
S2—C22	1.7710 (14)	C12—C13	1.4018 (19)
S2—C2	1.8050 (17)	C13—C14	1.381 (2)
O1—N5	1.2208 (16)	C13—H13	0.9500
O2—N5	1.2226 (16)	C14—C15	1.385 (2)
N1—N2	1.2793 (17)	C14—H14	0.9500
N1—C11	1.4028 (17)	C15—C16	1.374 (2)
N2—C3	1.3754 (17)	C15—H15	0.9500
N3—C3	1.3009 (17)	C16—H16	0.9500
N3—N4	1.3148 (17)	C21—C26	1.387 (2)
N4—C21	1.4029 (17)	C21—C22	1.399 (2)
N4—H4	0.8800	C22—C23	1.391 (2)
N5—C3	1.4720 (18)	C23—C24	1.380 (2)
C1—H1A	0.9800	C23—H23	0.9500
C1—H1B	0.9800	C24—C25	1.379 (2)
C1—H1C	0.9800	C24—H24	0.9500
C2—H2A	0.9800	C25—C26	1.385 (2)
C2—H2B	0.9800	C25—H25	0.9500
C2—H2C	0.9800	C26—H26	0.9500
			
C12—S1—C1	102.72 (7)	C11—C12—S1	121.56 (10)
C22—S2—C2	100.39 (7)	C14—C13—C12	121.98 (13)
N2—N1—C11	115.05 (11)	C14—C13—H13	119.0
N1—N2—C3	113.46 (11)	C12—C13—H13	119.0
C3—N3—N4	119.26 (12)	C13—C14—C15	121.09 (14)
N3—N4—C21	119.70 (11)	C13—C14—H14	119.5
N3—N4—H4	120.1	C15—C14—H14	119.5
C21—N4—H4	120.1	C16—C15—C14	118.39 (14)
O1—N5—O2	123.76 (13)	C16—C15—H15	120.8
O1—N5—C3	117.59 (12)	C14—C15—H15	120.8
O2—N5—C3	118.65 (11)	C15—C16—C11	121.74 (13)
S1—C1—H1A	109.5	C15—C16—H16	119.1
S1—C1—H1B	109.5	C11—C16—H16	119.1
H1A—C1—H1B	109.5	C26—C21—C22	120.22 (13)
S1—C1—H1C	109.5	C26—C21—N4	121.02 (13)
H1A—C1—H1C	109.5	C22—C21—N4	118.76 (12)
H1B—C1—H1C	109.5	C23—C22—C21	118.61 (13)
S2—C2—H2A	109.5	C23—C22—S2	119.38 (12)
S2—C2—H2B	109.5	C21—C22—S2	122.01 (10)
H2A—C2—H2B	109.5	C24—C23—C22	121.13 (14)
S2—C2—H2C	109.5	C24—C23—H23	119.4
H2A—C2—H2C	109.5	C22—C23—H23	119.4
H2B—C2—H2C	109.5	C25—C24—C23	119.69 (14)
N3—C3—N2	134.15 (13)	C25—C24—H24	120.2
N3—C3—N5	113.05 (11)	C23—C24—H24	120.2
N2—C3—N5	112.77 (11)	C24—C25—C26	120.40 (15)
C16—C11—N1	113.16 (12)	C24—C25—H25	119.8
C16—C11—C12	120.20 (12)	C26—C25—H25	119.8
N1—C11—C12	126.61 (13)	C25—C26—C21	119.93 (15)
C13—C12—C11	116.57 (13)	C25—C26—H26	120.0
C13—C12—S1	121.87 (11)	C21—C26—H26	120.0
			
C11—N1—N2—C3	−179.60 (11)	C12—C13—C14—C15	0.2 (2)
C3—N3—N4—C21	176.56 (13)	C13—C14—C15—C16	−1.6 (2)
N4—N3—C3—N2	1.0 (2)	C14—C15—C16—C11	1.6 (2)
N4—N3—C3—N5	−176.84 (12)	N1—C11—C16—C15	−178.69 (13)
N1—N2—C3—N3	1.7 (2)	C12—C11—C16—C15	−0.4 (2)
N1—N2—C3—N5	179.48 (11)	N3—N4—C21—C26	−1.4 (2)
O1—N5—C3—N3	161.94 (13)	N3—N4—C21—C22	178.64 (13)
O2—N5—C3—N3	−17.90 (19)	C26—C21—C22—C23	−1.7 (2)
O1—N5—C3—N2	−16.36 (18)	N4—C21—C22—C23	178.25 (13)
O2—N5—C3—N2	163.80 (13)	C26—C21—C22—S2	179.03 (12)
N2—N1—C11—C16	−173.42 (12)	N4—C21—C22—S2	−1.06 (19)
N2—N1—C11—C12	8.4 (2)	C2—S2—C22—C23	108.63 (13)
C16—C11—C12—C13	−0.93 (19)	C2—S2—C22—C21	−72.07 (13)
N1—C11—C12—C13	177.12 (13)	C21—C22—C23—C24	0.8 (2)
C16—C11—C12—S1	179.31 (11)	S2—C22—C23—C24	−179.83 (12)
N1—C11—C12—S1	−2.65 (19)	C22—C23—C24—C25	0.5 (2)
C1—S1—C12—C13	−7.47 (13)	C23—C24—C25—C26	−0.9 (3)
C1—S1—C12—C11	172.28 (12)	C24—C25—C26—C21	0.1 (3)
C11—C12—C13—C14	1.0 (2)	C22—C21—C26—C25	1.2 (2)
S1—C12—C13—C14	−179.22 (11)	N4—C21—C26—C25	−178.72 (15)

### Hydrogen-bond geometry (Å, °)

**Table:**

*D*—H···*A*	*D*—H	H···*A*	*D*···*A*	*D*—H···*A*
N4—H4···S2	0.88	2.60	3.0248 (13)	110.
N4—H4···N1	0.88	1.99	2.6229 (16)	128.
C2—H2B···O1^i^	0.98	2.36	3.253 (2)	151.
C25—H25···O2^ii^	0.95	2.45	3.1901 (19)	134.

Symmetry codes: (i) −*x*, *y*+1/2, −*z*+1/2; (ii) −*x*+2, −*y*, −*z*+1.
